# Preventing falls among older fallers: study protocol for a two-phase pilot study of the multicomponent LIVE LiFE program

**DOI:** 10.1186/s13063-018-3114-5

**Published:** 2019-01-03

**Authors:** Marianne Granbom, Lindy Clemson, Laken Roberts, Melissa D. Hladek, Safiyyah M. Okoye, Minhui Liu, Cynthia Felix, David L. Roth, Laura N. Gitlin, Sarah Szanton

**Affiliations:** 1 Center for Innovative Care in Aging, Johns Hopkins School of Nursing, 525 N. Wolfe St, Baltimore, MD 21205 USA; 2 Department of Health Sciences, Lund University, Lund, Sweden; 3 The University of Sydney, 75 East Street, Lidcombe, NSW 2141 Australia; 4 Johns Hopkins Bloomberg School of Public Health, 615 N Wolfe St, Baltimore, MD 21205 USA; 5 Center on Aging and Health, Johns Hopkins University, 2024 E. Monument Street, Baltimore, MD 21205 USA; 6 College of Nursing and Health Professions, Drexel University, 1601 Cherry Street, Philadelphia, PA 19102 USA

**Keywords:** Older adults, Prevention, Lifestyle-integrated exercise, Occupational therapy, Community-dwelling, Near falls, Home hazards, Home visit, Vision screening, Medication review

## Abstract

**Background:**

Falls reflect sentinel events in older adults, with significant negative consequences. Although fall risk factors have been identified as intrinsic (e.g., muscle weakness, balance problems) and extrinsic (e.g., home hazards), most prevention programs target only intrinsic factors. We present the rationale and design of a home-based multicomponent fall prevention program—the LIVE LiFE program—for community-living older adults. The program adapts and expands the successful Lifestyle Intervention Functional Exercise (LiFE) program by adding home safety, vision contrast screening, and medication review. The specific aims of the study are to (1) adapt the LiFE program to a US context and expand it into a multicomponent program (LIVE LiFE) addressing intrinsic and extrinsic fall risks, (2) examine feasibility and acceptability, and (3) estimate program impact on multiple outcome measures to prepare for an efficacy trial.

**Methods:**

The study involves two phases: an open-label pilot, followed by a two-group, single-blinded randomized pilot trial. Eligible participants are community-living adults 70+ years reporting at least one injurious fall or two non-injurious falls in the previous year. Participants are randomized in a 2:1 ratio to the program group (LIVE LiFE, *n* = 25) or the control group (written fall risk assessment, *n* = 12). The open-label pilot participants (*n* = 3) receive the program without randomization and are assessed based on their experience, resulting in a stronger emphasis on the participant’s personal goals being integrated into LIVE LiFE. Fall risk and balance outcomes are assessed by the Timed Up and Go and the 4-Stage Balance Test at 16 weeks. Additional outcomes are incidence of falls and near falls, falls efficacy, fear of falling, number of home hazards, and medications assessed at 16 weeks. Incidence of falls and near falls, program adherence, and satisfaction are assessed again at 32 weeks.

**Discussion:**

By expanding and adapting the evidence-based LiFE program, our study will help us understand the feasibility of conducting a multicomponent program and estimate its impact on multiple outcome measures. This will support moving forward with an efficacy trial of the LIVE LiFE program for older adults who are at risk of falling.

**Trial registration:**

ClinicalTrials.gov, NCT03351413. Registered on 22 November 2017.

**Electronic supplementary material:**

The online version of this article (10.1186/s13063-018-3114-5) contains supplementary material, which is available to authorized users.

## Background

Falls reflect a sentinel event severely impacting the health and quality of life of older adults as well as resulting in healthcare costs. As the demographic profile of countries continues to reflect dramatic age transformation, the risk of falling will persist. Each year in the USA, ~ 29 million (28.7%) adults 65 years of age and older fall, leading to 7 million injuries [1]. In 2015, the estimated annual cost of fatal falls in the USA was $637.5 billion and for non-fatal falls, $31.3 billion, a 3% increase in costs over 3 years [2]. Both injurious and non-injurious falls affect older adults’ quality of life. Fear of another fall can limit daily life, with good reason, as a fall predicts having future falls [3–5]. Also, fall risk factors are usually multiple and interrelated. Substantial evidence shows that intrinsic factors such as advanced age, poor balance, gait problems, history of falls, certain medications, polypharmacy, and vision impairment as well as extrinsic factors such as home hazards and their combination can cause falls in older adults [3, 6–8]. Near falls—stumbles or trips that do not result in a fall—also predict fall risk [9].

Although it is recognized that there are multiple contributors to falls and fall risk in older adults, in most cases the programs designed to prevent and reduce falls only address one or two risk factors. Which ones that are addressed and how they are addressed vary greatly.

Balance and lower-limb strength exercise are well known to effectively reduce falls in older adults [10], but a limitation of fall prevention studies that rely on exercise is the challenge of adherence and long-term use of strategies by older adults. A program addressing those challenges is the Lifestyle Intervention Functional Exercise (LiFE) program [11], which integrates strength and balance activities within routine daily activities. In an Australian trial, LiFE increased balance and leg strength and reduced the number of falls in community-living older adults with high risk of falls.

However, to address both intrinsic and extrinsic fall risks, multicomponent programs are needed. A 2012 Cochrane review showed that exercise programs, home safety, medication reduction, and treatments for vision problems reduce the risk of falling [10]. A recent systematic review and meta-analysis showed that multicomponent programs that combine exercise with vision and home safety assessments and/or treatments are associated with reductions in injurious falls [12]; however, more evidence is needed to prove clinically meaningful effects of multicomponent programs on community-living older adults with high risk of falls [13].

Although there is evidence of effective strategies to reduce falls, implementation of community-based fall prevention programs in the USA has been limited and varies by state and county [14]. In addition to the limited availability of evidence-based fall prevention programs, practical barriers such as transportation, effort, costs, and dislike of group activities may prevent people from attending classes when they are available in their communities—particularly for those older people at higher risk of falls [15]. Tailored home-based programs have potential advantages; e.g., they can address home safety and home hazards and they do not require transportation to a gym or group-based training. However, most importantly, tailored home-based programs have been shown to be more effective and better adhered to than non-tailored programs [16].

We present the rationale and design of a two-step pilot study of a home-based, multicomponent fall prevention program for community-living older adults (LIVE LiFE), building from the successful LiFE program [11] and adding home safety, vision contrast screening, and medication review. LIVE LiFE addresses intrinsic and extrinsic risk factors in a tailored home-based program delivered by an occupational therapist (OT). The specific aims of the study are to (1) adapt the LiFE program to a US context and expand it into a multicomponent program (LIVE LiFE) addressing intrinsic and extrinsic fall risks, (2) examine feasibility and acceptability, and (3) estimate program impact on multiple outcome measures to prepare for a larger efficacy trial.

## Methods/design

### Study design

**Fig. 1:**
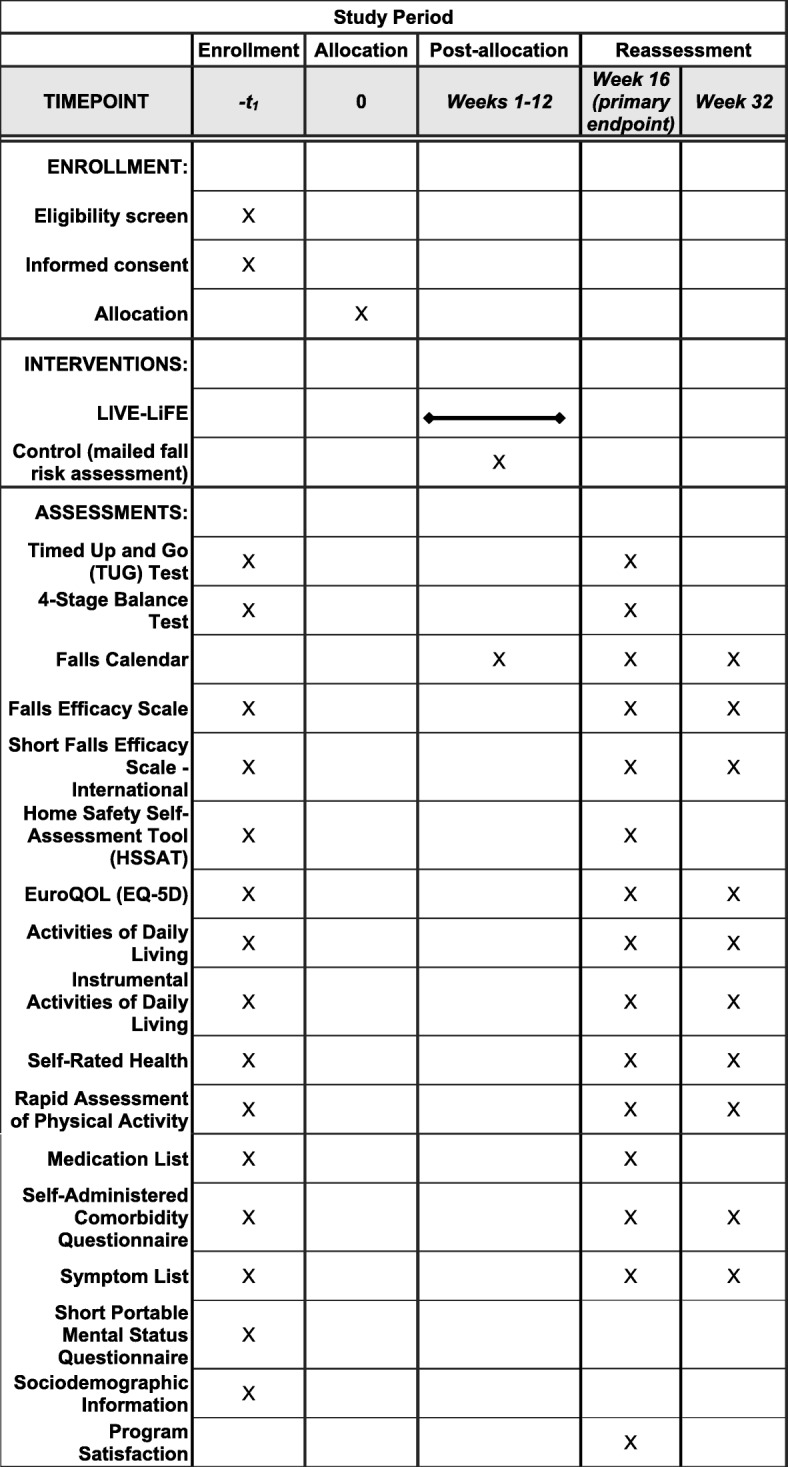
Overview of LIVE LiFE enrollment, interventions, and assessments adapted from the Standard Protocol Items: Recommendations for Interventional Trials (SPIRIT) figure

This study is a single-site study conducted in Baltimore, Maryland, USA. We have combined an open-label pilot and a single-blind randomized pilot trial in a two-phase protocol. First, we conducted an open-label pilot study to refine content and optimize the delivery of the program in which the first three participants received the program (*n* = 3). The second phase is a single-blind randomized pilot trial (*n* = 37). Participants are randomly assigned on a 2:1 ratio to the LIVE LiFE group or the control group. The program is conducted by OTs in the home of the study participants with referral to optometrist and primary care provider when indicated. The program group receives the 12-week LIVE LiFE program, and the control group receives a written fall risk assessment using data from baseline measures. During home visits, trained research assistants conduct the data collection at baseline, at 16 weeks, which is the primary endpoint of the trial, and at 32 weeks to determine whether program gains are maintained. The study will be implemented and reported in line with Standard Protocol Items: Recommendations for Interventional Trials (SPIRIT) guidelines [17] (Fig. 1 and Additional file 1).

### Study population

The study population is community-living people 70 years of age or older from the Baltimore, Maryland, area who report two or more falls or one injurious fall in the previous year. Exclusion criteria are moderate to severe cognitive limitations based on the Short Portable Mental Status Questionnaire [18], no conversational English, inability to stand, or being a resident in a residential care facility. Also, older adults are excluded if they report being hospitalized more than three times in the last year or report a terminal diagnosis (less than 1 year expected survival or receiving active cancer treatment). The inclusion criteria are broad to reflect older adults aging with a fall history; however, we exclude individuals who are too ill to participate or who have cognitive impairment, because the program involves follow-through on prescribed exercises.

### Recruitment

We recruit from multiple sources including flyers at libraries, pharmacies, and senior centers in the area and also individuals who were not eligible for study participation in other local ongoing fall prevention trials. Interested older adults contact the study office and are screened for eligibility over the phone and confirmed in person. If the participant is eligible and interested in partaking, the research assistant obtains written informed consent and conducts the baseline data collection.

### Randomization and blinding

Because the program involves multiple components, we anticipate some variability in aspects of implementation. Consequently, we employ a 2:1 randomization ratio of program vs. control condition such that a greater number of participants will be randomized to the program. We hypothesize that this would allow us to more easily observe variability in the program and facilitate implementation for planning future research, while still providing similar power for the primary between-group comparisons. Within 48 h of the home visit, an affiliated researcher not involved in data collection or in the program randomizes the participant using a computer-based assignment scheme. The project manager sends the assignment by mail to the participant and notifies the OT. The OT contacts the participant within 2 weeks to schedule the first appointment. Research assistants blinded to group allocation conduct data collection and data entry at follow-up.

### Sample size

We assume an attrition rate of 13% during the 16-week follow-up based on our experience with previous clinical trials of similar duration and intensity and in the same community [19]. Based on our experiences piloting multicomponent home-based programs [20], we will recruit 40 participants, including participants to the open-label pilot (*n* = 3) and participants randomized to LIVE LiFE (*n* = 25) or to the control group (*n* = 12).

### The LIVE LiFE program

The overarching theoretical framework for the program is Verbrugge and Jette’s Disablement Process, which stresses that both intrinsic (body and mind) and extrinsic (environment) factors can contribute to a process of disablement [21]. Also, we draw upon the Szanton-Gill resilience model [22], which suggests that intervening on both individual and environmental factors simultaneously may lead to more lasting program benefits. The program is also built on theories suggesting that lifestyle changes that are built upon the individual’s established daily routines and habits are more likely to lead to sustainable changes [23–25].

Based on what we learned in the open-label phase, the home-based multicomponent fall prevention program included goal clarification, balance and strength training integrated into daily habits, home safety assessment, home hazards removal, vision screening, and medication review (see Table 1). The 12-week program is led by an OT and contains eight home visits and two booster calls from the OT and two visits from a handyman (described in detail below).

**Table 1:** Dimension, components, and treatment approaches of the LIVE LiFE program

Dimensions	Fall risk reduction component	Origin	Treatment approach
Intrinsic	Personal goals to improve motivation and adherence to the program	The Purpose in Life literature [47], incorporated based on results from open-label phase of the current study	The OT asks about personal goals and explicitly directs the program toward the goals (e.g., to walk to church, to spend time with grandchildren)
Intrinsic	Exercise to improve balance and strength	The LiFE program [11]	Over the course of 8 home visits the OT coaches the participant to incorporate balance and strength activities in daily life activities, for example, tandem walking when walking through the hallway or standing on one leg when working in the kitchen
Intrinsic	Medication review for medication safety	Beers report [29]	Pharmacist reviews scanned list of medications that potentially increase the risk of falls and writes a letter with recommendations to the participant to share with their primary care provider
Intrinsic	Vision contrast screening to optimize vision	Mars [28]	At-home vision contrast screening provided by the OT using the Mars tool [28]. The results and recommendations are written in a letter to the participant to share with the primary care provider or an optometrist
Extrinsic	Home safety: reduce fall hazards in and around the home	Results on home modification interventions, e.g., the CAPABLE program [19]	The OT assesses home safety and provides smaller modifications (e.g., night lights, non-skid strips for the bathtub). Creates a prioritized list of modifications for handyman (e.g., installing grab bars in bathroom, stabilizing shaky banisters, and fixing holes in floors). Handyman installs items up to $500/participant

The LiFE program is the basis of LIVE LiFE and is the balance and strength component. It is a home-based exercise program developed in Australia for community-living older adults at risk of falling. LiFE is a standardized program effective in reducing falls and recommended by the Centers for Disease Control and Prevention (CDC) [26]. The content of the LiFE program has been published [11, 27]. Briefly, eight balance (including static and dynamic) and six strength (hip, knee, and ankle) domains form the basis of these exercises. The older adult chooses movements that improve balance and strength to integrate into daily/household/leisure activities. With help from the OT, participants plan opportunities to practice during the day, which increases adoption and continuation of the exercise program, as it becomes a part of daily life. The activities are tailored to the capacity of the person based on assessments the OT conducts on falls history, functional in-home balance and strength tests, and contextual resources and limitations. The activities are upgraded safely over time. Adherence to the exercise program is recorded daily by the participants and reviewed at each session by the OT.

#### Expansion of the LiFE program

The home safety component includes an assessment of safety features in the home and ≤ US $500 to address home hazards such as lack of banisters and grab bars, holes in the floor, dim lighting, or slippery floors. After the home safety assessment, the OT suggests possible solutions and provides small adaptations such as nightlights or fluorescent tape applied to slippery steps. For more extensive home hazards, the OT and the participant brainstorm and agree on prioritized modifications and repairs (e.g., installing grab bars in the bathroom or banisters in the stairway). The OT sends a work order to a licensed handyman. When the handyman obtains the order, she or he usually visits the participant twice, first to plan and then to install the adaptations and repairs. This component of the program is provided by Civic Works, an AmeriCorps site in Baltimore, MD.

For the vision screening component, the OT does a contrast sensitivity vision screening with the Mars Letter Contrast Sensitivity Test [28]. To reflect the usual visual conditions, participants are asked to keep the room lit as usual during the assessment. The Mars results and recommendations are written in a letter and given to the participant to provide to their primary care provider. If the results do not meet the recommended threshold for older adults provided in the Mars manual, participants are referred to an optometrist for an extended vision examination.

For the medication review component, a pharmacologist specializing in older adults reviews a list of the prescribed and over-the-counter drugs collected at the baseline visit and provides simple recommendations in a letter to the participants to share with their primary care provider. The review is based on the updated Beers criteria for potentially inappropriate medication use in older adults [29]. We specifically target medications known to increase the risk of falls (e.g., medications that target blood pressure or blood sugar, mood stabilizers), the total number of medications, combinations of medications, doses, and frequencies.

### The control group

Participants in the control group receive a written fall risk assessment by mail based on their baseline scores. They are also sent the “Stay Independent” brochure on how to reduce the risk of falling published by the CDC [30].

### Data collection and management

Data collectors receive project-specific training in the measures used. Data are collected via encrypted, password-protected tablet computers at participants’ homes at baseline and follow-up at 16 weeks. A second follow-up is completed by phone interview at 32 weeks. All data are uploaded to the online data entry and management system software REDCap. The study implements several data quality features offered by REDCap, including range checks, branching logic, and calculated fields to minimize data entry errors. Study visits are audio-recorded with patient consent for the study data manager to listen to in the event an error is identified. Audio recordings are destroyed at the end of the study after all data quality checks have been completed. The participants receive a $25 gift card after each completed data collection ($75 in total).

### Outcomes

For feasibility and acceptability measures, we collect data on refusal to be enrolled after being explained the study, and study retention rates. The OT documents the number (dose) and duration (intensity) of sessions to capture intervention delivery. Documentation on adherence to strength and balance training is provided through an activity log completed daily by the participant and reviewed weekly by the OT. At the end of the program, the OT follows up with the participants as to whether or not they had received the vision and medication referral, whether they have acted on them, and whether the handyman portion of the program has been completed. For acceptability, we collect data on program satisfaction at baseline and at 16 and 32 weeks.

To prepare for an efficacy trial, we explore multiple outcome measures to estimate program impact.

For fall risk and balance, we use the Timed Up and Go (TUG) test [31] and the 4-Stage Balance Test [32]. The TUG test measures time in seconds for the participants to get up from a chair, walk 3 m, turn, return to the chair, and sit down. The TUG test has good reliability for trained data collectors [33] and predicts falls in older adults with lower functioning, such as those with a history of falls [34]. The 4-Stage Balance test is a test of static balance [32]. The participant holds for increasingly more challenging balance positions: side-by-side, semi-tandem (the instep of one foot touching the big toe of the other foot), tandem (one foot in front of the other), and balancing on one leg. If an older adult cannot hold the tandem (third position) for 10 s, they are considered at high risk for falls [35].

Number of falls and near falls are recorded daily by the participants using an investigator-developed monthly falls calendar. A fall is defined as a person unintentionally coming to rest on the ground, floor, or other lower level [36]. A near fall is defined as a fall that is initiated but arrested by support, for example, from the wall, railing, or another person [37]. Participants mail the completed calendars at the end of each month using preaddressed, stamped envelopes. A research assistant phones participants who do not return a calendar to ascertain whether they had fallen. When a fall or near fall is marked on the calendar, the research assistant phones and asks follow-up questions on where the fall or near fall took place and whether injuries occurred.

Because fear of falling is central to decreasing falls risk and is considered on the causal pathway, we used multiple measures to learn more about this important concept. Falls efficacy is measured by the 10-item Falls Efficacy Scale (FES), which asks the participant to rate from 0 to 10 their confidence in doing ten activities in or outside the home without falling [38]. Fear of falling is measured with the Short FES-International (FES-I) scale to assess the participant’s concern about falling when doing seven activities in and around the home. Both the FES and the FES-I are known to mediate fall prevention improvement [39, 40]. We also ask the participants three single-item questions about their perceptions on fear of falling, including: Are you concerned about falling?, Are you afraid of falling?, and Do you fear falling?

To assess reduction in home hazards, we use the Home Safety Self-Assessment Tool (HSSAT) checklist—a 67-item checklist filled out by the data collector during a home tour with the participant. The house tour includes front and back entrances, hallway, living room, kitchen, bathroom, bedroom, basement/laundry room, stairways, and garage [41]. The HSSAT is a standardized assessment with sufficient reliability and validity to assess home hazards [41, 42].

For the medication review component, we assess use of medications as a simple count and listing of the participant’s prescribed and over-the-counter drugs. Participants are also asked whether they have discussed their medications and any associated fall-related concerns with their doctor in the last year (yes/no). The participants are asked whether they have recieved a vision check-up in the last year and whether they use bifocals (yes/no).

We consider possible confounding by assessing difficulties in Activities of Daily Living (ADL) with the Katz ADL Index on eight basic (e.g., eating, bathing) and eight instrumental ADLs (e.g., washing laundry, managing finances) [43, 44]. The participants report if they had no difficulty and did not need help the prior month (0), had difficulty but did not need help (1), or did need help regardless of difficulty (2). Health-related quality of life is measured by the EuroQol questionnaire (EQ-5D) [45]. Self-rated health is assessed by asking the participants how they would rate their health in general, from poor (5) to excellent (0). Mobility device use is assessed by asking the participants whether they have used mobility devices indoors and outdoors in the last week and month.

### Fidelity plan

The fidelity plan, based on the National Institutes of Health (NIH) Behavior Change Consortium [46], addresses fidelity through design (distinct program based on theory), training (using established LiFE program training, home safety training, and program manual), delivery (reminder calls the night before sessions), engagement (records of home sessions by date and duration), and receipt (completing checklists on program engagement). To assure enactment, participants in the LIVE LiFE group demonstrate the last session’s exercises to the OT on the following session and show the logs of activities. Sessions are audio-recorded, and study team members review 10% of the sessions. We have bi-weekly meetings with the OT and principal investigator (PI), in which case presentations are provided and evaluated in terms of fidelity to intervention delivery. In case of a need for protocol amendments or any adverse events during the study, direct handling will be dealt with by the project coordinator under PI supervision and reported to the Institutional Review Board (IRB).

### Analysis

#### Open-label pilot

When the open-label phase participants had completed more than half of the intervention sessions, a study team member interviewed them over the phone. They were asked open-ended questions on the content and delivery of the balance and strength component, home safety component, medication review component, vision screening component, and documentation of the program. The PI interviewed the OT on the same components. The open-label phase revealed that participants did not always feel intrinsically motivated to do the prescribed exercises. Subsequently, drawing upon the health promotion literature on Purpose in Life [47], we adapted the program to have the OT ask the participant about their own goals, such as spending time with family, cooking dinner, or going to outside activities. Then, based on the participant’s self-identified goals, we adapted the participants´ LIVE LiFE exercise log to prominently display the goal they had chosen for themselves. This allowed the participants to see their exercise activities as connected to or in view of their own goals rather than as something the OT was asking them to do as homework.

#### Feasibility, acceptability, and responses to primary and secondary outcomes

For feasibility and acceptability of the program, we will examine percentages of people who stay in each arm of the study, and conduct descriptive correlational analyses of the association between the program compliance and demographic and participant health variables. Also, the feasibility of delivering the program will be assessed by exploring the OTs’ documentation of dose and duration. For program compliance analyses, we will distinguish non-compliance with the program from attrition or loss to follow-up, i.e., missing data. We will use the participant satisfaction data to examine the acceptability of the program. To examine whether the program group continues with the exercise component beyond the end of the program, we will use data collected up until 32 weeks to inspect frequency distributions derived from the adherence tracking logs and examine correlations with demographic and other factors to explore predictors of adherence. Also, we will continue to collect fall diaries and examine group differences in falls and near falls up through 32 weeks after the onset of the program.

All analyses of primary and secondary outcomes will follow the intention-to-treat principle: all participants will be counted in their assigned study group once the assignment has been made. We will then perform a sensitivity analysis by excluding the open-label pilot participants (*n* = 3) from the remaining LIVE LiFE group participants. For the primary aim, the outcome will be improvement in the TUG and the 4-Stage Balance test between baseline and 16 weeks. Effect sizes will be estimated based on Cohen’s *D*. We will decide on a parametric or non-parametric testing approach after reviewing sample sizes and skewness of data. Otherwise, our general analytic approach for the primary outcome will be standard analyses of covariance (ANCOVAs) on the 16-week outcome data, with the baseline value of each respective outcome variable serving as a covariate. For the secondary aims, analyses will use chi-square tests or logistic regression analyses to examine group differences for data collected at 16 and 32 weeks.

## Discussion

Reviews on fall prevention programs suggest that multicomponent programs are more effective than single-component programs [10, 12]. With this study, we seek to adapt and expand a balance and strength program proven previously to be effective to address a wider range of intrinsic and extrinsic fall risks than in its original form. LIVE LiFE benefits from adapting components already proven effective and currently in use in other settings from the LiFE program, the Community Aging in Place—Advancing Better Living for Elders (CAPABLE) program, and the Beers report [11, 19, 29]. The purpose of this pilot trial is to provide a foundation for a larger definitive trial.

Adding components to LiFE increases its complexity; thus, testing feasibility is essential at this stage of the program development. As a first step to enhance feasibility, we conducted an open-label pilot that provided valuable insights as to how to improve program content and its delivery. The LIVE LiFE program has the potential to increase adherence to the program and increase motivation by focusing on life goals, by integrating the exercise component into daily habits at home, and by making the home environment safer. With these aspects, the older adult is more likely to continue the exercise program and stay active after the trial has ended, which is imperative to have long-lasting effects on fall reduction [11, 16].

### Protocol version and trial status

The protocol is version number 1. We started recruitment in October 2017 and will end data collection in spring 2019. The trial was registered at ClinicalTrials.gov on 22 November^,^ 2017, identifier NCT03351413.

## Additional file

Additional file 1: SPIRIT checklist. (DOC 142 kb)

## Abbreviations

ADL: Activities of daily living
ANCOVA: Analysis of covariance
CAPABLE: Community Aging in Place—Advancing Better Living for Elders
CDC: Centers for Disease Control and Prevention
EQ-5D: EuroQol questionnaire
FES: Falls Efficacy Scale
FES-I: Short Falls Efficacy Scale-International
HSSAT: Home Safety Self-Assessment Tool
LiFE: Lifestyle Intervention Functional Exercise
OT: Occupational therapist
TUG: Timed Up and Go (test)
